# Structural properties and isomorphism theorems for Cayley digraphs of full transformation semigroups with respect to Green's equivalence classes

**DOI:** 10.1016/j.heliyon.2023.e12976

**Published:** 2023-01-13

**Authors:** Nuttawoot Nupo, Yanisa Chaiya

**Affiliations:** a Department of Mathematics, Faculty of Science, Khon Kaen University, Khon Kaen, 40002, Thailand; b Department of Mathematics and Statistics, Faculty of Science and Technology, Thammasat University, Pathum Thani, 12120, Thailand

**Keywords:** Cayley digraph, Full transformation semigroup, Green's relations, Isomorphism theorem, Connectedness

## Abstract

Let T(X) be the full transformation semigroup on a nonempty set *X*. In this paper, the Cayley digraphs of T(X) with connection sets *L* and *R*, the Green's equivalence classes of T(X) according to the Green's relations L and R, are investigated. Furthermore, their connectedness properties are characterized. In addition, the isomorphism theorems for Cayley digraphs of T(X) are also presented.

## 1 Introduction and preliminaries

Let *A* be a nonempty subset of a semigroup *S*. The *Cayley digraph* Cay(S,A) *of a semigroup S relative to A* is known as a digraph with vertex set *S* and arc set consisting of ordered pairs (x,xa)∈S×S for some a∈A. The set *A* will be called a *connection set* of Cay(S,A). Cayley digraphs of semigroups have been extensively investigated, see, for example [1], [2], [3], [4], [5]. One of semigroups, which is widely considered, is the transformation semigroup since any semigroup can be embedded into a transformation semigroup on an appropriate set. This would be a general result why the transformation semigroup is interesting to study. Several authors studied structural properties of Cayley graphs of transformation semigroups, see, for example [6], [7], [8], [9], [10], [11].

We now provide important preliminaries for this paper. Throughout the paper, all sets are considered to be finite. Let *X* be a nonempty set and T(X) the set of all full transformations from *X* into itself. It is well known that T(X) is a regular semigroup under the composition of functions. Throughout this paper, we write the functions on the right, that is, for a composition *αβ*, *α* is applied first. We now introduce the definition of Green's relations L and R for which L-classes and R-classes are considered to be connections sets of Cayley digraphs of T(X).

Let *S* be a semigroup and $S^{1}$ denote a semigroup obtained from *S* by adjoining an identity if *S* has no identity and $S^{1}=S$ if it already contains an identity. The following definitions are due to Green. For any a,b∈S, define

$$a\;L\;b\text{ if and only if }S^{1}a=S^{1}b,$$

or equivalently, aLb if and only if a=xb,b=ya for some $x,y\in S^{1}$. Dually, define

$$a\;R\;b\text{ if and only if }aS^{1}=bS^{1},$$

or equivalently, aRb if and only if a=bx,b=ay for some $x,y\in S^{1}$. Moreover, define

aHb if and only if aLb and aRb,

that is, H=L∩R. Note that the relations L,R and H are all equivalence relations on *S*. Furthermore, for each a∈S, denote by $L_{a},R_{a}$ and $H_{a}$ the L-class, R-class and H-class containing *a*, respectively.

Let α∈T(X). The notation *xα* means the image of *x* under *α* and Xα={xα:x∈X} is called the *image* of *α*. In addition, $\pi_{\alpha }$ stands for the set $\left\{x\alpha^{-1}:x\in X\alpha \right\}$. Especially, for the transformation *α* such that $X\alpha =\{a_{1},a_{2},\ldots ,a_{n}\}$, we usually write this *α* as

$$\alpha =\left(\begin{array}{cccc} A_{1} & A_{2} & \ldots & A_{n}\\ a_{1} & a_{2} & \ldots & a_{n} \end{array}\right)$$

and take as understood that $a_{i}\alpha^{-1}=A_{i}$ for all i=1,2,…,n, that is, $\pi_{\alpha }=\{A_{1},A_{2},\ldots ,A_{n}\}$ is a partition of *X*. The following theorem shows the characterizations of Green's relations L and R on T(X).

Theorem 1.1 [12] *For any* α,β∈T(X) *,*

1. αLβ *if and only if* Xα=Xβ *,*
2. αRβ *if and only if* $\pi_{\alpha }=\pi_{\beta }$ *.*

In this paper, the Cayley digraphs of the full transformation semigroup T(X), where *X* is a finite nonempty set, with the connection sets *L* and *R*, the Green's equivalence classes of T(X) have been constructed for studying their structural properties. Furthermore, the isomorphism theorems of the Cayley digraphs have been presented.

## 2 Cayley digraphs of T(X) with respect to L-classes

This section provides structural properties of Cayley digraphs of T(X) with respect to L-classes and *X* is a nonempty finite set. Hereafter, we denote by $T_{n}$ the full transformation semigroup T(X) when X={1,2,…,n} for some n∈N. Recall that, for any $\lambda \in T_{n}$, the L-class containing *λ* is $L_{\lambda }=\{\alpha :X\alpha =X\lambda \}$. Hence, each L-class of $T_{n}$ is of the form $L_{A}=\{\alpha \in T_{n}:X\alpha =A\}$, where ∅≠A⊆X. Note that a digraph *D* is said to be *totally complete* if E(D)=V(D)×V(D). We now start with some characterizations of Cayley digraphs of $T_{n}$ with certain L-classes.

Theorem 2.1 *Let*$\lambda \in T_{n}$*and*$L_{\lambda }$*be an*L*-class containing λ. Let*$\Gamma :=\mathrm{Cay}(T_{n},L_{\lambda })$*. If*$\delta \in L_{\lambda }$*, then an induced subdigraph*$\Gamma [H_{\delta }]$*of* Γ *is totally complete where* $H_{\delta }$ *is an* H*-class of* $T_{n}$ *containing δ.*

Proof Let $\delta \in L_{\lambda }$ and $\alpha ,\beta \in H_{\delta }$. Since δLλ, we have Xδ=Xλ. Then we can write

$$\lambda =\left(\begin{array}{cccc} A_{1} & A_{2} & \ldots & A_{k}\\ a_{1} & a_{2} & \ldots & a_{k} \end{array}\right)\text{ and }\delta =\left(\begin{array}{cccc} B_{1} & B_{2} & \ldots & B_{k}\\ a_{1} & a_{2} & \ldots & a_{k} \end{array}\right).$$

Further, since αHδHβ, we obtain that Xα=Xδ=Xβ and $\pi_{\alpha }=\pi_{\delta }=\pi_{\beta }$. Thus we can write

$$\alpha =\left(\begin{array}{cccc} B_{1} & B_{2} & \ldots & B_{k}\\ a_{\phi (1)} & a_{\phi (2)} & \ldots & a_{\phi (k)} \end{array}\right)\text{ and }\beta =\left(\begin{array}{cccc} B_{1} & B_{2} & \ldots & B_{k}\\ a_{\varphi (1)} & a_{\varphi (2)} & \ldots & a_{\varphi (k)} \end{array}\right),$$

where *ϕ* and *φ* are bijections on the set {1,2,…,k} and $\left\{a_{\phi (i)}:i=1,2,\ldots ,k\}=\{a_{1},a_{2},\ldots ,a_{k}\}=\{a_{\varphi (i)}:i=1,2,\ldots ,k\right\}$. Let $\gamma \in T_{n}$ be defined by

$$\gamma =\left(\begin{array}{cccc} a_{\phi (1)} & a_{\phi (2)} & \ldots & a_{\phi (k)}\cup C\\ a_{\varphi (1)} & a_{\varphi (2)} & \ldots & a_{\varphi (k)} \end{array}\right),$$

where $C=X\setminus \{a_{\phi (i)}:i=1,2,\ldots ,k\}$. Clearly, Xγ=Xλ which implies that $\gamma \in L_{\lambda }$. Moreover, we obtain that β=αγ. Thus (α,β)∈E(Γ). From $\alpha ,\beta \in H_{\delta }$, we have $\left(\alpha ,\beta )\in E(\Gamma [H_{\delta }]\right)$. This completes the proof. □

In order to present the next theorem, we need to note that $S_{n}$ means the group of all permutations in $T_{n}$ with identity $id_{X}$ where |X|=n. We now illustrate an example of the characterization for the Cayley digraph of $T_{n}$ with respect to the connection set $S_{n}$ which is one of L-classes of $T_{n}$.

**Figure 1:**
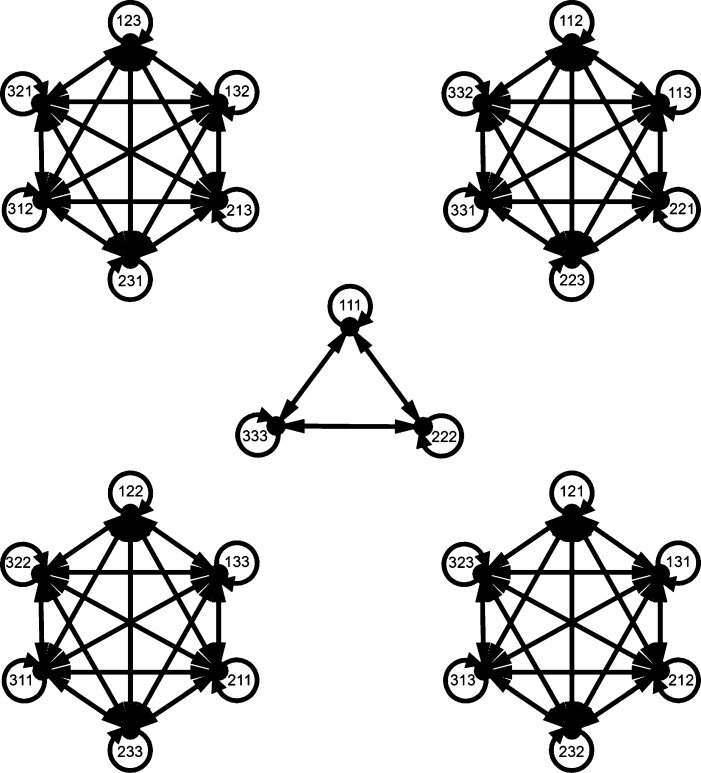
$\mathrm{Cay}(T_{3},S_{3})$.

Example 2.2 Let X={1,2,3}. Then $|T_{3}|=27$. For convenience, each element $\left(\begin{array}{ccc} 1 & 2 & 3\\ x & y & z \end{array}\right)$ in $T_{3}$ will be written as “*xyz*”. Then $S_{3}=\{123,132,213,231,312,321\}$ and thus $\mathrm{Cay}(T_{3},S_{3})$ is shown in Fig. 1. We can observe that the set of all vertices in the same component forms an R-class of $T_{3}$. Hence $\mathrm{Cay}(T_{3},S_{3})$ is the disjoint union of totally complete subdigraphs which each of them is induced by an R-class of $T_{3}$.

Theorem 2.3 *Let L be an*L*-class of*$T_{n}$*and*$\Gamma :=\mathrm{Cay}(T_{n},L)$*. Then* Γ *is the disjoint union of totally complete induced subgraphs* Γ[R]*, where R is an* R*-class of* $T_{n}$*, if and only if* $L=S_{n}$*.*

Proof Let $L=S_{n}$ and *R* be an R-class of $T_{n}$. Let α,β∈R. Then $\pi_{\alpha }=\pi_{\beta }$ and we can write

$$\alpha =\left(\begin{array}{cccc} A_{1} & A_{2} & \ldots & A_{k}\\ a_{1} & a_{2} & \ldots & a_{k} \end{array}\right)\text{ and }\beta =\left(\begin{array}{cccc} A_{1} & A_{2} & \ldots & A_{k}\\ b_{1} & b_{2} & \ldots & b_{k} \end{array}\right).$$

We can observe that |Xα|=k=|Xβ|. It follows that |X∖Xα|=n−k=|X∖Xβ|. Let $X\setminus X\alpha =\{c_{i}:i=1,2,\ldots ,n-k\}$ and $X\setminus X\beta =\{d_{i}:i=1,2,\ldots ,n-k\}$. Define $\gamma \in T_{n}$ by

$$\gamma =\left(\begin{array}{cccccccc} a_{1} & a_{2} & \ldots & a_{k} & c_{1} & c_{2} & \ldots & c_{n-k}\\ b_{1} & b_{2} & \ldots & b_{k} & d_{1} & d_{2} & \ldots & d_{n-k} \end{array}\right).$$

Thus $\gamma \in S_{n}$. Furthermore, β=αγ which yields that (α,β)∈E(Γ). Hence Γ[R] forms a totally complete subdigraph of Γ.

Next, let (α,β) be an arbitrary arc of Γ. Then there exists $\lambda \in L=S_{n}$ in which β=αλ. It follows that $\alpha =\beta \lambda^{-1}$ such that $\lambda^{-1}\in S_{n}$. That means αRβ. Thus *α* and *β* belong to the same R-class of $T_{n}$. Therefore, Γ is the disjoint union of totally complete subdigraphs Γ[R] where *R* is an R-class of $T_{n}$.

Conversely, assume that the condition holds. Let α∈L. It is easy to verify that $\left(id_{X},\alpha )\in E(\Gamma \right)$. By the assumption, we have $\alpha \in R_{id_{X}}$ which implies that $\pi_{\alpha }=\pi_{id_{X}}$. Thus $\alpha \in S_{n}$. On the other hand, let $\alpha \in S_{n}$. Then $\pi_{\alpha }=\pi_{id_{X}}$ and hence $\alpha \;R\;id_{X}$. Since a subdigraph of Γ induced by an R-class is totally complete, we have $\left(id_{X},\alpha )\in E(\Gamma [R])\subseteq E(\Gamma \right)$. Hence $\alpha =id_{X}\beta$ for some $\beta \in L=S_{n}$, that is, $\alpha =\beta \in S_{n}$. Consequently, $L=S_{n}$, as required. □

For each a∈X, denote by $\chi_{a}$ the constant map $\left(\begin{array}{c} X\\ a \end{array}\right)\in T_{n}$. It is easy to see that $\left\{\chi_{a}\}=\{\alpha \in T_{n}:X\alpha =\{a\}\right\}$ is an L-class of $T_{n}$. The following theorem is the characterization of Cayley digraphs with respect to L-classes of such type.

Theorem 2.4 *Let L be an* L *-class of* $T_{n}$ *,* $\Gamma :=\mathrm{Cay}(T_{n},L)$ *and* a∈X *. Then* $L=\{\chi_{a}\}$ *if and only if* $E(\Gamma )=\{(\alpha ,\chi_{a}):\alpha \in T_{n}\}$ *.*

Proof Let $L=\{\chi_{a}\}$ and $\alpha \in T_{n}$. Since $\chi_{a}=\alpha \chi_{a}$ where $\chi_{a}\in L$, we have $\left(\alpha ,\chi_{a})\in E(\Gamma \right)$. We next let (λ,δ)∈E(Γ). Then $\delta =\lambda \chi_{a}$ where $\chi_{a}\in L$. Hence $\delta =\chi_{a}$. Therefore, $E(\Gamma )=\{(\alpha ,\chi_{a}):\alpha \in T_{n}\}$.

Conversely, assume that $E(\Gamma )=\{(\alpha ,\chi_{a}):\alpha \in T_{n}\}$. Clearly, $\left(id_{X},\chi_{a})\in E(\Gamma \right)$ which leads to $\chi_{a}=id_{X}\lambda$ where λ∈L. Thus $\chi_{a}=\lambda \in L$. Next, suppose that there exists α∈L in which $\alpha \neq \chi_{a}$. This gives $\left(id_{X},\alpha )\in E(\Gamma \right)$ which contradicts to the assumption. So we conclude that $L=\{\chi_{a}\}$. □

Let $\Gamma :=\mathrm{Cay}(T_{n},L)$. For each $\alpha \in T_{n}$, we note that

$$N(\alpha )=\{\beta \in T_{n}:(\alpha ,\beta )\in E(\Gamma )\}.$$

If (α,β)∈E(Γ), then *β* will be called a *neighbour* of $\alpha \in T_{n}$ and the set N(α) is called the *neighbourhood* of $\alpha \in T_{n}$. Next, we characterize the neighbourhoods of constant maps and permutations in $T_{n}$.

Lemma 2.5 *Let*$L_{A}$*be a connection set of* Γ *and* a∈X*. Then* $N(\chi_{a})=\{\chi_{b}:b\in A\}$*.*

Proof Let a∈X. To show that $N(\chi_{a})=\{\chi_{b}:b\in A\}$, we first let $\alpha \in N(\chi_{a})$. Then $\left(\chi_{a},\alpha )\in E(\Gamma \right)$, that is, $\alpha =\chi_{a}\beta$ for some $\beta \in L_{A}$. It follows that $\alpha =\chi_{a\beta }$ where aβ∈A. On the other hand, let b∈A. We prove that $\left(\chi_{a},\chi_{b})\in E(\Gamma \right)$. For convenience, let $A=\{b,a_{1},a_{2},\ldots ,a_{k}\}$ and X∖A=C. Let I={1,2,…,k}. We have two cases to consider.

**Case 1:**$a=a_{l}$ for some l∈I. Then define

$$\alpha ={\left(\begin{array}{ccc} \{a\}\cup C & b & a_{i}\\ b & a & a_{i} \end{array}\right)}_{i\in I\setminus \{l\}}.$$

Hence $\alpha \in L_{A}$ and $\chi_{b}=\chi_{a}\alpha$ which yields $\left(\chi_{a},\chi_{b})\in E(\Gamma \right)$.

**Case 2:**a=b or a∉A. We then define

$$\beta ={\left(\begin{array}{cc} \{b\}\cup C & a_{i}\\ b & a_{i} \end{array}\right)}_{i\in I}.$$

Thus $\beta \in L_{A}$ and $\chi_{b}=\chi_{b}\beta =\chi_{a}\beta$ and so $\left(\chi_{a},\chi_{b})\in E(\Gamma \right)$.

From the above two cases, we have $\chi_{b}\in N(\chi_{a})$. □

Lemma 2.6 *Let*$L_{A}$*be a connection set of* Γ *in which* 1≤|A|≤n−1*. If* $\alpha \in S_{n}$*, then* $N(\alpha )=L_{A}$*.*

Proof Let $\alpha \in S_{n}$. We first show that $L_{A}$ contains N(α). Let β∈N(α). Then (α,β)∈E(Γ), that is, β=αγ for some $\gamma \in L_{A}$. We need to prove that Xβ=A. Clearly, Xβ⊆Xγ=A. Next, let a∈A. Thus there exists b∈X such that bγ=a. Since *α* is surjective, there exists c∈X in which cα=b. Hence cβ=c(αγ)=(cα)γ=bγ=a which implies that a∈Xβ and thus A⊆Xβ. Therefore, Xβ=A and then $\beta \in L_{A}$.

For the other containment, we let $\beta \in L_{A}$. Since *α* is surjective, we have Xα=X. Define *γ* by (xα)γ=xβ for every x∈X. Since *α* is bijective, *γ* is well-defined. This implies that $\gamma \in T_{n}$. We now show that (α,β)∈E(Γ). For each x∈X, xαγ=(xα)γ=xβ. Thus αγ=β. From the definition of *γ*, it is obvious that Xγ⊆Xβ=A. Let a∈A. There exists x∈X in which xβ=a. Hence (xα)γ=xβ=a, that is, a∈Xγ. We have A⊆Xγ which yields that Xγ=A. Therefore, $\gamma \in L_{A}$. Consequently, (α,β)∈E(Γ) and then β∈N(α). □

Next, we provide necessary and sufficient conditions for being arcs of Γ which also give the characterization of neighbours of *α* where *α* is neither a permutation nor a constant map in $T_{n}$. To purpose the results, we need the following definition.

Let A and B be families of sets. We say that A *refines* B if for each A∈A, there exists B∈B in which A⊆B.

Lemma 2.7 *Let* $\alpha ,\beta \in T_{n}$ *. Then* $\beta \in \alpha T_{n}$ *if and only if* $\pi_{\alpha }$ *refines* $\pi_{\beta }$ *.*

Proof Let $\alpha ,\beta \in T_{n}$. Assume that $\beta \in \alpha T_{n}$. Then β=αγ for some $\gamma \in T_{n}$. Let $A\in \pi_{\alpha }$. Thus $A=x\alpha^{-1}$ for some x∈Xα. For each y∈A, we have yα=x and so xγ=(yα)γ=y(αγ)=yβ∈Xβ. Hence $(x\gamma )\beta^{-1}\in \pi_{\beta }$. We now prove that *A* is contained in $(x\gamma )\beta^{-1}$. Let a∈A. It follows that aα=x and thus aαγ=xγ. That means aβ=xγ which implies that $a\in (x\gamma )\beta^{-1}$. Therefore, $A\subseteq (x\gamma )\beta^{-1}\in \pi_{\beta }$. Consequently, $\pi_{\alpha }$ refines $\pi_{\beta }$.

Conversely, assume that $\pi_{\alpha }$ refines $\pi_{\beta }$. For each x∈Xα, there exists $x^{\prime }\in X$ in which $x^{\prime }\alpha =x$. Define $\gamma \in T_{n}$ as follows:

$$x\gamma =\left\{ \begin{array}{cc} x^{\prime }\beta , & x\in X\alpha ,\\ x\alpha , & x\notin X\alpha . \end{array}\right.$$

Hence, for each x∈X, we obtain that $x\alpha \gamma =(x\alpha )\gamma ={\left(x\alpha \right)}^{\prime }\beta$. Since xα∈Xα, we have ${\left(x\alpha \right)}^{\prime }\alpha =x\alpha$. Then there exists $P\in \pi_{\alpha }$ in which $\{{\left(x\alpha \right)}^{\prime },x\}\subseteq P$. Since $\pi_{\alpha }$ refines $\pi_{\beta }$, there exists $P^{\prime }\in \pi_{\beta }$ such that $P\subseteq P^{\prime }$ and so $\{{\left(x\alpha \right)}^{\prime },x\}\subseteq P^{\prime }$. It follows that ${\left(x\alpha \right)}^{\prime }\beta =x\beta$. Hence αγ=β and thus $\beta \in \alpha T_{n}$, as required. □

Theorem 2.8 *Let*$L_{A}$*be a connection set of* Γ *and* $\alpha ,\beta \in T_{n}$*. Then* (α,β)∈E(Γ) *if and only if the following statements hold:*

1. $\pi_{\alpha }$*refines*$\pi_{\beta }$*,*
2. Xβ⊆A*,*
3. |X∖Xα|≥|A∖Xβ|*.*

Proof Assume that (α,β)∈E(Γ). Then there exists $\gamma \in L_{A}$ such that β=αγ. By Lemma 2.7, we obtain that $\pi_{\alpha }$ refines $\pi_{\beta }$. Moreover, we have Xβ=Xαγ⊆Xγ=A. As the fact that β=αγ, the number of sets in $\pi_{\gamma }$ containing elements of *Xα* is |Xβ|. Hence the number of sets in $\pi_{\gamma }$ which do not contain elements of *Xα* is exactly |A∖Xβ|. Therefore, |X∖Xα|≥|A∖Xβ|.

Conversely, assume that the conditions hold. We will show that there exists $\gamma \in L_{A}$ such that β=αγ. Since |X∖Xα|≥|A∖Xβ|, there exists a surjective function σ:X∖Xα→A∖Xβ. Moreover, for each x∈Xα, there will be $x^{\prime }\in X$ such that $x^{\prime }\alpha =x$. Define $\gamma \in T_{n}$ by

$$x\gamma =\left\{ \begin{array}{cc} x^{\prime }\beta , & x\in X\alpha ,\\ x\sigma , & x\notin X\alpha . \end{array}\right.$$

Let x∈X. Then xα∈Xα and thus there exists ${\left(x\alpha \right)}^{\prime }\in X$ such that ${\left(x\alpha \right)}^{\prime }\alpha =x\alpha$. Hence ${\left(x\alpha \right)}^{\prime },x\in P$ for some $P\in \pi_{\alpha }$. Since $\pi_{\alpha }$ refines $\pi_{\beta }$, we obtain that ${\left(x\alpha \right)}^{\prime }\beta =x\beta$. Therefore, $x\alpha \gamma =(x\alpha )\gamma ={\left(x\alpha \right)}^{\prime }\beta =x\beta$ which implies that β=αγ and (Xα)γ=Xβ. Since Xγ=(Xα)γ∪(X∖Xα)γ=Xβ∪(A∖Xβ)=A, $\gamma \in L_{A}$. Consequently, (α,β)∈E(Γ). □

In order to purpose the following theorem, we need to define certain notation. For a nonempty subset *S* of $T_{n}$, the neighbourhood of *S* in $T_{n}$ is $N(S)=\underset{\alpha \in S}{⋃}N(\alpha )$ where N(α) is the neighbourhood of *α* in $T_{n}$.

Theorem 2.9 *Let*$L_{A}$*be a connection set of* Γ *such that* ∅≠A⊊X*. If* |A|=k *and* |X|=n*, then* $N(L_{A})$ *is the union of* L*-classes* $L_{B}$ *where* B⊆A *and* |B|≥2k−n*.*

Proof Let ∅≠A⊊X be such that |A|=k and |X|=n. Then k≤n−1. Let $\alpha \in N(L_{A})$. Thus α∈N(β) for some $\beta \in L_{A}$ and hence α=βγ for some $\gamma \in L_{A}$. We obtain that Xα=Xβγ⊆Xγ=A. Again from α=βγ, we can conclude by Theorem 2.8 that |X∖Xβ|≥|A∖Xα|. This implies that n−k≥k−|Xα| and so |Xα|≥2k−n. Therefore,

$$\alpha \in L_{X\alpha }\subseteq \underset{B\subseteq A}{⋃}L_{B},$$

where |B|≥2k−n. Next, let $\alpha \in L_{D}$ for some D⊆A and |D|≥2k−n. For convenience, we may assume that $A=\{a_{1},a_{2},\ldots ,a_{k}\}$. We now consider the following two cases.

**Case 1:**D=A. Then Xα=A. So we can write

$$\alpha =\left(\begin{array}{cccc} A_{1} & A_{2} & \ldots & A_{k}\\ a_{1} & a_{2} & \ldots & a_{k} \end{array}\right).$$

Let C=X∖A and define

$$\beta =\left(\begin{array}{cccc} \{a_{1}\}\cup C & a_{2} & \ldots & a_{k}\\ a_{1} & a_{2} & \ldots & a_{k} \end{array}\right).$$

Clearly, $\beta \in L_{A}$ and α=αβ. Thus (α,α)∈E(Γ), that is, $\alpha \in N(\alpha )\subseteq N(L_{A})$.

**Case 2:**D⊊A. Let $D=\{d_{1},d_{2},\ldots ,d_{t}\}$ and $A\setminus D=\{z_{1},z_{2},\ldots ,z_{k-t}\}$ be such that t<k and t≥2k−n. We can write *α* as follows:

$$\alpha =\left(\begin{array}{cccc} A_{1} & A_{2} & \ldots & A_{t}\\ d_{1} & d_{2} & \ldots & d_{t} \end{array}\right).$$

Since t<k<n, there exists $A_{j}$ such that $|A_{j}|\geq 2$ for some j∈{1,2,…,t}. Choose $a_{j}\in A_{j}$. Let $A_{j}^{\prime }=A_{j}\setminus \{a_{j}\}$. Since $|\{d_{s}\in D:s\neq j\}\cup \{d_{j}\}\cup (A\setminus D)|=k$, we can choose $b_{1},b_{2},\ldots ,b_{k-t}\in \overset{t}{\underset{j=1}{⋃}}A_{j}^{\prime }$ which are all distinct. Further, since t≥2k−n, we have n≥2k−t and thus n−k≥k−t. Now, choose $w_{1},w_{2},\ldots ,w_{k-t}\in X\setminus A$ and let $Y=X\setminus (A\cup \{w_{1},w_{2},\ldots ,w_{k-t}\})$. Define *β* and *γ* as follows:

$$\beta ={\left(\begin{array}{cccccc} A_{s} & A_{j}\setminus \{b_{1},b_{2},\ldots ,b_{k-t}\} & b_{1} & b_{2} & \ldots & b_{k-t}\\ d_{s} & d_{j} & z_{1} & z_{2} & \ldots & z_{k-t} \end{array}\right)}_{s\in \{1,2,\ldots t\}\setminus \{j\}}\text{ and }\;\gamma ={\left(\begin{array}{cccccc} d_{s} & \{d_{j}\}\cup T_{j} & w_{1} & w_{2} & \ldots & \{w_{k-t}\}\cup Y\\ d_{s} & d_{j} & z_{1} & z_{2} & \ldots & z_{k-t} \end{array}\right)}_{s\in \{1,2,\ldots t\}\setminus \{j\}},$$

where $T_{j}=\{z_{m}:b_{m}\in A_{j}\}$. Therefore, βγ=α where $\gamma \in L_{A}$. Consequently, $\alpha \in N(\beta )\subseteq N(L_{A})$. □

Now, we are ready to present characterizations for connectedness of the Cayley digraph $\Gamma :=\mathrm{Cay}(T_{n},L_{A})$ where $L_{A}$ is an L-class of $T_{n}$. If n=1, then Γ has only one vertex which is a trivial digraph. Hence, from now on, we will consider the Cayley digraph Γ of $T_{n}$ where n>1. Let u,v be two distinct vertices of a digraph *D*. Throughout the paper, the notation [u,v]*-semidipath* denotes the semidipath from *u* to *v*. Similarly, [u,v]*-dipath* denotes the dipath from *u* to *v*. The digraph *D* is called a *strongly connected* digraph if *D* contains a [u,v]-dipath. Moreover, *D* is called a *weakly connected* digraph if *D* contains a [u,v]-semidipath. The digraph *D* is called a *locally connected* digraph whenever a [u,v]-dipath exists in *D*, a [v,u]-dipath must exist in *D* as well. Further, *D* is called a *unilaterally connected* digraph if either a [u,v]-dipath or a [v,u]-dipath exists in *D*.

Theorem 2.10 *The Cayley digraph* Γ *is never strongly connected.*

Proof As we obtain by Lemma 2.5 that $N(\chi_{a})=\{\chi_{b}:b\in A\}$, there is no dipath from $\chi_{a}$ through to *α* in which |Xα|>1. This implies that Γ is not strongly connected. □

Theorem 2.11 Γ *is weakly connected if and only if* |A|≤n−1*.*

Proof If |A|=n, then $L_{A}=S_{n}$. By Theorem 2.3, we obtain that Γ is the disjoint union of totally complete induced subdigraphs Γ[R] where *R* is an R-class of $T_{n}$. It follows that Γ is not weakly connected.

Conversely, let |A|≤n−1. For convenience, let $A=\{a_{1},a_{2},\ldots ,a_{k}\}$ and b∉A. Further, let B=X∖(A∪{b}). Define $\beta_{1}\in T_{n}$ as follows:

$$\beta_{1}=\left(\begin{array}{cccc} A_{1} & A_{2} & \ldots & A_{k}\\ a_{1} & a_{2} & \ldots & a_{k} \end{array}\right).$$

Then $\beta_{1}\in L_{A}$. Moreover, for each i=2,3,…,k, we define $\beta_{i}$ by

$$\beta_{i}={\left(\begin{array}{ccc} \{a_{1},a_{i}\}\cup B & b & x\\ a_{1} & a_{i} & x \end{array}\right)}_{x\in A\setminus \{a_{1},a_{i}\}}.$$

It is not hard to verify that $\beta_{1}\beta_{2}\cdots \beta_{k}=\chi_{a_{1}}$ where $\beta_{i}\in L_{A}$ for 1≤i≤k. Now, let α,β be arbitrary vertices of Γ. We obtain that

$$\alpha \beta_{1}\beta_{2}\cdots \beta_{k}=\alpha \chi_{a_{1}}=\beta \chi_{a_{1}}=\beta \beta_{1}\beta_{2}\cdots \beta_{k}.$$

Hence Γ contains an $\left[\alpha ,\chi_{a_{1}}\right]$-dipath and a $\left[\beta ,\chi_{a_{1}}\right]$-dipath. Therefore, there is a weakly dipath joining between *α* and *β* in Γ. Consequently, Γ is weakly connected. □

Let $\beta \in T_{n}$. Note that $N^{-}(\beta )=\{\alpha \in T_{n}:(\alpha ,\beta )\in E(\Gamma )\}$.

Theorem 2.12 *The Cayley digraph* Γ *is never unilaterally connected.*

Proof Let $L_{A}$ be a connection set of Γ. For the case |A|=n, we can conclude by Theorem 2.3 that Γ is the disjoint union of totally complete induced subdigraphs Γ[R] where *R* is an R-class of $T_{n}$. It follows that Γ is not unilaterally connected. We next consider |A|≤n−1. Let $\alpha \in T_{n}$. In fact, |X(αμ)|≤|Xμ|≤n−1 for all $\mu \in L_{A}$. This implies that $\alpha \mu \notin S_{n}$ for all $\mu \in L_{A}$. Hence $N^{-}(\beta )=\emptyset$ for all $\beta \in S_{n}$. We can conclude that there is no any dipath joining between elements in $S_{n}$. Therefore, Γ is not unilaterally connected. □

Theorem 2.13 Γ *is locally connected if and only if* |A|=n*.*

Proof Assume that |A|=n. That means $L_{A}=S_{n}$. By Theorem 2.3, we can conclude that every component of Γ is totally complete. This clearly implies that Γ is locally connected.

Conversely, let Γ be locally connected. Let $\alpha \in L_{A}$ be a given element. Hence $\left(id_{X},\alpha )\in E(\Gamma \right)$. By the locally connectedness of Γ, there exists a dipath joining from *α* back to $id_{X}$ in Γ, say *P*. Thus *P* can be written as a sequence $\alpha ,\alpha_{1},\alpha_{2},\ldots ,\alpha_{k},id_{X}$ for some $\alpha_{i}\in T_{n}$ where i∈{1,2,…,k}. Then $id_{X}=\alpha_{k}\lambda_{1}=(\alpha_{k-1}\lambda_{2})\lambda_{1}=\ldots =(\alpha_{1}\lambda_{k})\lambda_{k-1}\cdots \lambda_{2}\lambda_{1}=\alpha =\lambda \lambda_{k}\lambda_{k-1}\cdots \lambda_{2}\lambda_{1}$ where $\lambda ,\lambda_{i}\in L_{A}$ for all i∈{1,2,…,k}. Since $\lambda \lambda_{k}\lambda_{k-1}\cdots \lambda_{2}\lambda_{1}\in T_{n}$ and by Lemma 2.7, we obtain that $\pi_{\alpha }$ refines $\pi_{id_{X}}$. This implies that $\alpha \in S_{n}$. Since $S_{n}$ is an L-class of $T_{n}$ containing *α*, we have $L_{A}=S_{n}$. Hence |A|=n. □

The last part of this section, we will present the sufficient condition for an isomorphism theorem of the Cayley digraph Γ with respect to the connection set $L_{A}$.

Theorem 2.14 *Let* $\Gamma_{1}$ *and* $\Gamma_{2}$ *denote* $\mathrm{Cay}(T_{n},L_{A_{1}})$ *and* $\mathrm{Cay}(T_{n},L_{A_{2}})$ *, respectively. If* $\left|A_{1}|=|A_{2}\right|$ *, then* $\Gamma_{1}≅\Gamma_{2}$ *.*

Proof Assume that $\left|A_{1}|=|A_{2}\right|$. Let $A_{1}\cap A_{2}=B$. Then $\left|A_{1}\setminus B|=|A_{2}\setminus B\right|$. Hence there is a bijection $g:A_{1}\setminus B\to A_{2}\setminus B$. Let α:X→X be defined by

$$x\alpha =\left\{ \begin{array}{cc} g(x), & x\in A_{1}\setminus B,\\ g^{-1}(x), & A_{2}\setminus B,\\ x, & \text{ otherwise}, \end{array}\right.$$

for all x∈X. Clearly, $\alpha \in T_{n}$ is also a bijection. Then we define $\varphi :T_{n}\to T_{n}$ as follows:

$$\varphi \left(\left(\begin{array}{cccc} A_{1} & A_{2} & \ldots & A_{k}\\ a_{1} & a_{2} & \ldots & a_{k} \end{array}\right)\right)=\left(\begin{array}{cccc} A_{1} & A_{2} & \ldots & A_{k}\\ a_{1}\alpha & a_{2}\alpha & \ldots & a_{k}\alpha \end{array}\right),$$

for all $\left(\begin{array}{cccc} A_{1} & A_{2} & \ldots & A_{k}\\ a_{1} & a_{2} & \ldots & a_{k} \end{array}\right)\in T_{n}$. It is not hard to verify that *φ* is a bijection. For each $\beta \in T_{n}$, we can see that $X\beta \subseteq A_{1}$ if and only if $X\varphi (\beta )\subseteq A_{2}$. Further, we have $\pi_{\varphi (\beta )}=\pi_{\beta }$ which implies that |Xβ|=|Xφ(β)|. Moreover, if $X\beta \subseteq A_{1}$, then $\left|A_{1}\setminus X\beta |+|X\beta |=|A_{1}|=|A_{2}|=|A_{2}|=|A_{2}\setminus X\varphi (\beta )|+|X\varphi (\beta )\right|$. Thus $\left|A_{1}\setminus X\beta |=|A_{2}\setminus X\varphi (\beta )\right|$. Therefore,

$$(\alpha ,\beta )\in E(\Gamma_{1})\Leftrightarrow \pi_{\alpha }\text{ refines }\pi_{\beta },X\beta \subseteq A_{1}\text{ and }|X\setminus X\alpha |\geq |A_{1}\setminus X\beta |\Leftrightarrow \pi_{\varphi (\alpha )}\text{ refines }\pi_{\varphi (\beta )},X\varphi (\beta )\subseteq A_{2}\text{ and }|X\setminus X\varphi (\alpha )|\geq |A_{2}\setminus X\varphi (\beta )|\Leftrightarrow (\varphi (\alpha ),\varphi (\beta ))\in E(\Gamma_{2}).$$

Consequently, $\Gamma_{1}≅\Gamma_{2}$. □

## 3 Cayley digraphs of T(X) with respect to R-classes

In this section, we study structural properties of Cayley digraphs Γ of $T_{n}$ where their connection sets are precisely R-classes of $T_{n}$. For each $\alpha \in T_{n}$, we recall that $\pi_{\alpha }=\{x\alpha^{-1}:x\in X\alpha \}$. Indeed, $\pi_{\alpha }$ forms a partition of *X*. Note that two elements $\alpha ,\beta \in T_{n}$ will be R-related if and only if $\pi_{\alpha }=\pi_{\beta }$, that is, the partitions of *X* induced by those two elements coincide. Given the partition $P=\{A_{1},A_{2},\ldots ,A_{k}\}$ of *X* where 1≤k≤n and let $R_{P}=\{\alpha \in T_{n}:\pi_{\alpha }=P\}$. Clearly, $R_{P}$ is an R-class of $T_{n}$. From now on, the set $R_{P}$ stands for the connection set of Γ.

Lemma 3.1 *Let*P*be any partition of X and*$R_{P}$*a connection set of* Γ*. If* $K=\{\chi_{a}:a\in X\}$*, then an induced subdigraph* Γ[K] *is totally complete.*

Proof Let $K=\{\chi_{a}:a\in X\}$ and $\chi_{a},\chi_{b}\in K$. For convenience, assume that $P=\{A_{1},A_{2},\ldots ,A_{t}\}$ for some t∈N. Without loss of generality, let $a\in A_{1}$. We then have the following two cases to consider.

**Case 1:**$b\in A_{1}$. We define $\alpha \in T_{n}$ as follows:

$$\alpha =\left(\begin{array}{cc} A_{1} & A_{i}\\ b & a_{i} \end{array}\right),$$

where $a_{i}\in A_{i}$ for all i∈{2,3,…,t} and $a_{i}\notin \{a,b\}$. Then $\alpha \in R_{P}$. Furthermore, we obtain that $\chi_{a}\alpha =\chi_{b}$ which leads to $\left(\chi_{a},\chi_{b})\in E(\Gamma \right)$.

**Case 2:**$b\notin A_{1}$. Assume, without loss of generality, that $b\in A_{2}$. We define $\alpha \in T_{n}$ as follows:

$$\alpha =\left(\begin{array}{ccc} A_{1} & A_{2} & A_{i}\\ b & a & a_{i} \end{array}\right),$$

where $a_{i}\in A_{i}$ for all i∈{3,4,…,t} and $a_{i}\notin \{a,b\}$. Then $\alpha \in R_{P}$. We can see that $\chi_{a}\alpha =\chi_{b}$ which implies that $\left(\chi_{a},\chi_{b})\in E(\Gamma \right)$.

From the above two cases, we conclude that Γ[K] is totally complete. □

Lemma 3.2 *If* $P=\{A_{1},A_{2},\ldots ,A_{k}\}$ *such that* 1≤k≤n−1 *, then* $\chi_{a}\in \langle R_{P}\rangle$ *for some* a∈X *where* $\langle R_{P}\rangle$ *is a subsemigroup of* $T_{n}$ *generated by* $R_{P}$ *.*

Proof Let $P=\{A_{1},A_{2},\ldots ,A_{k}\}$ be such that 1≤k≤n−1. Then there exists $A_{i}\in P$ in which $|A_{i}|\geq 2$. Without loss of generality, assume that $|A_{1}|\geq 2$. Let $a,b\in A_{1}$ and $a_{i}\in A_{i}$ for all i∈{2,3,…,k}. Now, for each j∈{2,3,…,k}, we define $\alpha_{j}\in T_{n}$ as follows:

$$\alpha_{j}=\left(\begin{array}{cccccccc} A_{1} & A_{2} & A_{3} & \ldots & A_{j} & A_{j+1} & \ldots & A_{k}\\ a & a_{2} & a_{3} & \ldots & b & a_{j+1} & \ldots & a_{k} \end{array}\right).$$

Hence $\alpha_{j}\in R_{P}$. Moreover, we see that

$$\alpha_{j}^{2}=\left(\begin{array}{ccccc} A_{1}\cup A_{j} & A_{2} & A_{3} & \ldots & A_{k}\\ a & a_{2} & a_{3} & \ldots & a_{k} \end{array}\right).$$

It is not hard to verify that $\alpha_{2}^{2}\alpha_{3}^{2}\cdots \alpha_{k}^{2}=\chi_{a}$, that is, $\chi_{a}\in \langle R_{P}\rangle$. □

We next present characterizations of Cayley digraphs Γ of $T_{n}$ with respect to connection sets $R_{P}$ where |P|=1 and |P|=n.

Theorem 3.3 *Let*P={X}*and*$R_{P}$*be a connection set of* Γ*. Then* $E(\Gamma )=\{(\alpha ,\chi_{a}):\alpha \in T_{n}\;\text{and}\;a\in X\}$*.*

Proof In this case, $R_{P}=\{\chi_{a}:a\in X\}$. For each $\alpha \in T_{n}$ and a∈X, we have $\chi_{a}=\alpha \chi_{a}$ where $\chi_{a}\in R_{P}$. Thus $\left(\alpha ,\chi_{a})\in E(\Gamma \right)$. On the other hand, let (α,β)∈E(Γ). Hence β=αγ for some $\gamma \in R_{P}$. That means $\gamma =\chi_{a}$ for some a∈X. Therefore, $\beta =\alpha \chi_{a}=\chi_{a}$. The proof is done. □

Note that |P|=n is equivalent to $R_{P}=S_{n}$ where $S_{n}$ is the group of all permutations in $T_{n}$. By Theorem 2.3, we have already proved the characterization of Γ in which the connection set is $S_{n}$. Consequently, the following result is obtained, directly.

Theorem 3.4 *Let*$R_{P}=S_{n}$*be a connection set of* Γ*. Then* Γ *is the disjoint union of totally complete induced subdigraphs* Γ[R] *where R is an* R*-class of* $T_{n}$*.*

Now, we provide the necessary and sufficient conditions of two elements in $T_{n}$ for being adjacent in Γ.

Theorem 3.5 *Let*$P=\{A_{1},A_{2},\ldots ,A_{k}\}$*be such that*1≤k≤n*and*$R_{P}$*a connection set of* Γ*. Let* $\alpha ,\beta \in T_{n}$*. Then* (α,β)∈E(Γ) *if and only if the following statements hold:*

1. $\pi_{\alpha }$*refines*$\pi_{\beta }$*,*
2. *for each*x,y∈X*,*xβ=yβ*if and only if*$x\alpha ,y\alpha \in A_{i}$*for some*i∈{1,2,…,k}*.*

Proof Let (α,β)∈E(Γ). Then β=αλ for some $\lambda \in R_{P}$. By Lemma 2.7, we obtain that $\pi_{\alpha }$ refines $\pi_{\beta }$. Let x,y∈X. We conclude that

$$x\beta =y\beta \Leftrightarrow x(\alpha \lambda )=y(\alpha \lambda )\Leftrightarrow (x\alpha )\lambda =(y\alpha )\lambda \Leftrightarrow x\alpha ,y\alpha \in A_{i},$$

for some i∈{1,2,…,k}.

Conversely, assume that the conditions hold. Since P is a partition of *X*, there exists i∈{1,2,…,k} such that $A_{i}\cap X\alpha \neq \emptyset$. Let $J=\{j\in \{1,2,\ldots ,k\}:A_{j}\cap X\alpha \neq \emptyset \}$. For each j∈J, choose $c_{j}\alpha \in A_{j}\cap X\alpha$. Further, for each t∈{1,2,…,k}∖J, choose $c_{t}\in X\setminus \{c_{j}\beta :j\in J\}$. Define λ:X→X as follows:

$$x\lambda =\left\{ \begin{array}{cc} c_{j}\beta , & x\in A_{j};j\in J,\\ c_{t}, & x\in A_{t};t\in \{1,2,\ldots ,k\}\setminus J. \end{array}\right.$$

Consider $c_{j}\alpha ,c_{j}^{\prime }\alpha \in A_{j}\cap X\alpha$, by the second condition in our assumption, we have $c_{j}\beta =c_{j}^{\prime }\beta$. Hence *λ* is well-defined, that is, $\lambda \in T_{n}$. We next show that $\lambda \in R_{P}$. Let x,y∈X. If $x,y\in A_{i}$ for some i∈{1,2,…,k}, then xλ=yλ by the definition of *λ*. Suppose that $x\in A_{i}$ and $y\in A_{j}$ for some i,j∈J and i≠j. Thus $x\lambda =c_{i}\beta \neq c_{j}\beta =y\lambda$ by the choice of $c_{i}$ and $c_{j}$. This implies that $\lambda \in R_{P}$. We now prove that β=αλ. Let x∈X. We have $x\alpha \in A_{j}$ for some j∈J and hence $x(\alpha \lambda )=(x\alpha )\lambda =c_{j}\beta$. Therefore, $x\alpha ,c_{j}\alpha \in A_{j}$ and by assumption, we conclude that $x\beta =c_{j}\beta$. Consequently, β=αλ which implies that (α,β)∈E(Γ), as required. □

The following proposition provides characterizations for neighbourhoods of constant maps and permutations in $T_{n}$.

Proposition 3.6 *Let*$P=\{A_{1},A_{2},\ldots ,A_{k}\}$*and*$R_{P}$*be a connection set of* Γ*.*

1. *If*a∈X*, then*$N(\chi_{a})=\{\chi_{b}:b\in X\}$*.*
2. *If*$\alpha \in S_{n}$*, then*$N(\alpha )=\{\beta \in T_{n}:\pi_{\beta }=\{A_{1}\alpha^{-1},A_{2}\alpha^{-1},\ldots ,A_{k}\alpha^{-1}\}\}$*.*

Proof To prove 1, let a∈X and $\beta \in N(\chi_{a})$. Then $\left(\chi_{a},\beta )\in E(\Gamma \right)$. By Theorem 3.5, we obtain that $\pi_{\chi_{a}}$ refines $\pi_{\beta }$. Thus $\pi_{\beta }=\{X\}=\pi_{\chi_{a}}$. Hence $\beta \in \{\chi_{b}:b\in X\}$ which implies that $N(\chi_{a})\subseteq \{\chi_{b}:b\in X\}$. On the other hand, we have by Lemma 3.1 that Γ[K] is totally complete, where $K=\{\chi_{b}:b\in X\}$, which yields $\left\{\chi_{b}:b\in X\}\subseteq N(\chi_{a}\right)$. Therefore, the statement 1 is completely proved.

For proving 2, we let $\alpha \in S_{n}$. Further, let β∈N(α). Thus there exists $\gamma \in R_{P}$ in which β=αγ. For each x,y∈X, we obtain that

$$x,y\in A_{i}\alpha^{-1}\text{ for some }A_{i}\in P\Leftrightarrow x\alpha ,y\alpha \in A_{i}\text{ for some }A_{i}\in P\Leftrightarrow x\beta =y\beta \text{ (by Theorem 3.5)}.$$

So we can conclude that $\pi_{\beta }=\{A_{1}\alpha^{-1},A_{2}\alpha^{-1},\ldots ,A_{k}\alpha^{-1}\}$. It is obvious that $\pi_{\alpha }$ refines $\pi_{\beta }$ since $\alpha \in S_{n}$. Moreover, for each x,y∈X, we have

$$x\beta =y\beta \Leftrightarrow x,y\in A_{i}\alpha^{-1}\text{ for some }i\in \{1,2,\ldots ,k\}\Leftrightarrow x\alpha ,y\alpha \in A_{i}\text{ for some }i\in \{1,2,\ldots ,k\}.$$

By Theorem 3.5, we conclude that (α,β)∈E(Γ), that is, β∈N(α). □

Next, we characterize the connectedness for Cayley digraphs Γ of $T_{n}$ (n>1) with respect to R-classes of $T_{n}$.

Theorem 3.7 *Let*P*be a partition of X and*$R_{P}$*a connection set of* Γ*. The Cayley digraph* Γ *is never unilaterally connected.*

Proof We first consider the case |P|=n, that is, $R_{P}=S_{n}$. By Theorem 2.3, we obtain that Γ is the disjoint union of totally complete induced subdigraphs Γ[R] where *R* is an R-class of $T_{n}$. Hence there is no any dipath joining between elements from different R classes. It follows that Γ is not unilaterally connected. We next consider |P|≤n−1. For each $\delta \in T_{n}$, we have |X(δλ)|≤|Xλ|<n for all $\lambda \in R_{P}$. Therefore, (δ,α)∉E(Γ) for all $\alpha \in S_{n}$. We can conclude that there is no any dipath joining between elements in $S_{n}$. Consequently, Γ is not unilaterally connected. □

As in the proof of Theorem 3.7, we know that Γ does not contain any dipath joining between two permutations in $S_{n}$. The following corollary is obtained, evidently.

Corollary 3.8 *Let*P*be a partition of X and*$R_{P}$*a connection set of* Γ*. The Cayley digraph* Γ *is never strongly connected.*

Theorem 3.9 *Let*P*be a partition of X and*$R_{P}$*a connection set of* Γ*. Then* Γ *is weakly connected if and only if* |P|≤n−1*.*

Proof If |P|=n, then $R_{P}=S_{n}$. We can conclude by Theorem 3.4 that Γ is not weakly connected. Conversely, let |P|≤n−1. For the case |P|=1, that is, $R_{P}=\{\chi_{a}:a\in X\}$, we have shown in Theorem 3.3 that $E(\Gamma )=\{(\alpha ,\chi_{a}):\alpha \in T_{n}\text{ and }a\in X\}$. This implies that Γ is weakly connected. Next, let 2≤|P|≤n−1. Let $\alpha \in T_{n}$. We will prove that Γ contains an $\left[\alpha ,\chi_{a}\right]$-dipath for some a∈X. By Lemma 3.2, we have $\chi_{a}\in \langle R_{P}\rangle$ for some a∈X. Thus there exists $\alpha_{1},\alpha_{2},\ldots ,\alpha_{t}\in R_{P}$ for some t∈N such that $\chi_{a}=\alpha_{1}\alpha_{2}\cdots \alpha_{t}$. Consider $\alpha \alpha_{1}\alpha_{2}\cdots \alpha_{t}=\alpha \chi_{a}=\chi_{a}$, the sequence $\alpha ,\alpha \alpha_{1},\alpha \alpha_{1}\alpha_{2},\ldots ,\alpha \alpha_{1}\alpha_{2}\cdots \alpha_{t-1},\chi_{a}$ forms an $\left[\alpha ,\chi_{a}\right]$-dipath in Γ. Therefore, for each $\beta ,\gamma \in T_{n}$, there exist a $\left[\beta ,\chi_{a}\right]$-dipath and a $\left[\gamma ,\chi_{b}\right]$-dipath in Γ for some a,b∈X. Furthermore, we have by Lemma 3.1 that Γ[K] is totally complete where $K=\{\chi_{a}:a\in X\}$ which leads to $\left(\chi_{a},\chi_{b})\in E(\Gamma \right)$. Hence the $\left[\beta ,\chi_{a}\right]$-dipath, the arc $\left(\chi_{a},\chi_{b}\right)$ and the $\left[\gamma ,\chi_{b}\right]$-dipath form a [β,γ]-semidipath in Γ. Therefore, Γ is weakly connected, as required. □

Note that $|A|=n\Leftrightarrow L_{A}=S_{n}=R_{P}\Leftrightarrow |P|=n$. Hence the following theorem can be obtained, directly, by Theorem 2.13.

Theorem 3.10 *Let*P*be a partition of X and*$R_{P}$*a connection set of* Γ*. Then* Γ *is locally connected if and only if* |P|=n*.*

Finally, we present the sufficient condition for an isomorphism theorem of Cayley digraphs of $T_{n}$ with respect to R-classes. Let $P=\{A_{1},A_{2},\ldots ,A_{k}\}$ be a partition of *X* such that $|A_{i}|=m_{i}$ for all i=1,2,…,k and $m_{1}\leq m_{2}\leq \ldots \leq m_{k}$. The sequence $m_{1},m_{2},\ldots ,m_{k}$ will be called the *degree sequence of* P.

Theorem 3.11 *Let* $P_{1}=\{A_{1},A_{2},\ldots ,A_{k}\}$ *and* $P_{2}=\{B_{1},B_{2},\ldots ,B_{k}\}$ *be partitions of X. If* $P_{1}$ *and* $P_{2}$ *have the same degree sequence, then* $\mathrm{Cay}(T_{n},R_{P_{1}})≅\mathrm{Cay}(T_{n},R_{P_{2}})$ *.*

Proof Let $\Gamma_{1}$ and $\Gamma_{2}$ denote $\mathrm{Cay}(T_{n},R_{P_{1}})$ and $\mathrm{Cay}(T_{n},R_{P_{2}})$, respectively. Further, assume that $P_{1}$ and $P_{2}$ have the same degree sequence. Thus $\left|A_{i}|=|B_{i}\right|$ for all i=1,2,…,k. Then, for each i=1,2,…,k, there exists a bijective function $f_{i}:A_{i}\to B_{i}$. Let λ:X→X be defined by $x\lambda =f_{i}(x)$ if $x\in A_{i}$. Hence $\lambda \in T_{n}$ is also a bijection. For each $\alpha \in T_{n}$, we define $\alpha^{⁎}:X\to X$ by $x\alpha^{⁎}=x(\alpha \lambda )$ for all x∈X, that is, $\alpha^{⁎}=\alpha \lambda$. So, for each x,y∈X, we obtain that

$$x\alpha =y\alpha \Leftrightarrow x(\alpha \lambda )=y(\alpha \lambda )\Leftrightarrow x\alpha^{⁎}=y\alpha^{⁎}.$$

This also implies that $\pi_{\alpha }=\pi_{\alpha^{⁎}}$. Let $\varphi :T_{n}\to T_{n}$ be defined by $\varphi (\alpha )=\alpha^{⁎}$ for all $\alpha \in T_{n}$. Since *λ* is a bijection, it follows that *φ* is a bijection. Moreover, for each $\alpha ,\beta \in T_{n}$, we have

$$\begin{array}{ccc} \left(\alpha ,\beta )\in E(\Gamma_{1}\right) & \Leftrightarrow & \pi_{\alpha }\text{ refines }\pi_{\beta }\text{ and for each }x,y\in X,\\ & & \left(x\beta =y\beta \Leftrightarrow x\alpha ,y\alpha \in A_{i}\text{ for some }i\in \{1,2,\ldots ,k\}\right)\\ & \Leftrightarrow & \pi_{\alpha^{⁎}}\text{ refines }\pi_{\beta^{⁎}}\text{ and for each }x,y\in X,\\ & & \left(x\beta^{⁎}=y\beta^{⁎}\Leftrightarrow x(\alpha \lambda ),y(\alpha \lambda )\in B_{i}\text{ for some }i\in \{1,2,\ldots ,k\}\right)\\ & \Leftrightarrow & \pi_{\varphi (\alpha )}\text{ refines }\pi_{\varphi (\beta )}\text{ and for each }x,y\in X,\\ & & \left(x\varphi (\beta )=y\varphi (\beta )\Leftrightarrow x\varphi (\alpha ),y\varphi (\alpha )\in B_{i}\text{ for some }i\in \{1,2,\ldots ,k\}\right)\\ & \Leftrightarrow & (\varphi (\alpha ),\varphi (\beta ))\in E(\Gamma_{2}). \end{array}$$

Consequently, $\Gamma_{1}≅\Gamma_{2}$. □

## 4 Conclusions

In this research, we have constructed the Cayley digraphs of full transformation semigroups whose connection sets are Green's equivalence classes L and R, respectively. To obtain their structural properties, the characterizations of being arcs in Cayley digraphs have been provided. Moreover, their connectedness have been investigated. Indeed, the connectedness property of Cayley digraphs with respect to L-classes and R-classes are rather resembled. Furthermore, their isomorphism theorems have also been presented.

## CRediT authorship contribution statement

**Yanisa Chaiya, Nuttawoot Nupo:** Conceived and designed the experiments; Performed the experiments; Analyzed and interpreted the data; Contributed reagents, materials, analysis tools or data; Wrote the paper.

## Declaration of Competing Interest

The authors declare no conflict of interest.

## Data Availability

No data was used for the research described in the article.
